# Circulating Apolipoprotein E Concentration and Cardiovascular Disease Risk: Meta-analysis of Results from Three Studies

**DOI:** 10.1371/journal.pmed.1002146

**Published:** 2016-10-18

**Authors:** Reecha Sofat, Jackie A. Cooper, Meena Kumari, Juan P. Casas, Jacqueline P. Mitchell, Jayshree Acharya, Simon Thom, Alun D. Hughes, Steve E. Humphries, Aroon D. Hingorani

**Affiliations:** 1 Centre for Clinical Pharmacology, Division of Medicine, University College London, London, United Kingdom; 2 Cardiovascular Genetics Group, Institute of Cardiovascular Science, University College London, London, United Kingdom; 3 Institute for Social and Economic Research, University of Essex, Colchester, United Kingdom; 4 Farr Institute of Health Informatics, Faculty of Population Health Sciences, University College London, United Kingdom; 5 International Centre for Circulatory Health, Hammersmith Hospital, Imperial College London, London, United Kingdom; 6 Cardiometabolic Phenotyping Group, Institute of Cardiovascular Science, University College London, London, United Kingdom; 7 Genetic Epidemiology Group, Institute of Cardiovascular Science, University College London, United Kingdom; University of Oxford, UNITED KINGDOM

## Abstract

**Background:**

The association of *APOE* genotype with circulating apolipoprotein E (ApoE) concentration and cardiovascular disease (CVD) risk is well established. However, the relationship of circulating ApoE concentration and CVD has received little attention.

**Methods and Findings:**

To address this, we measured circulating ApoE concentration in 9,587 individuals (with 1,413 CVD events) from three studies with incident CVD events: two population-based studies, the English Longitudinal Study of Ageing (ELSA) and the men-only Northwick Park Heart Study II (NPHSII), and a nested sub-study of the Anglo-Scandinavian Cardiac Outcomes Trial (ASCOT). We examined the association of circulating ApoE with cardiovascular risk factors in the two population-based studies (ELSA and NPHSII) and the relationship between ApoE concentration and coronary heart disease and stroke in all three studies. Analyses were carried out within study, and, where appropriate, pooled effect estimates were derived using meta-analysis. In the population-based samples, circulating ApoE was associated with systolic blood pressure (correlation coefficient 0.08, *p <* 0.001, in both ELSA and NPHSII), total cholesterol (correlation coefficient 0.46 and 0.34 in ELSA and NPHSII, respectively; both *p <* 0.001), low-density lipoprotein cholesterol (correlation coefficient 0.30 and 0.14, respectively; both *p <* 0.001), high-density lipoprotein (correlation coefficient 0.16 and −0.14, respectively; both *p <* 0.001), and triglycerides (correlation coefficient 0.43 and 0.46, respectivly; both *p <* 0.001). In NPHSII, ApoE concentration was additionally associated with apolipoprotein B (correlation coefficient 0.13, *p =* 0.001) and lipoprotein(a) (correlation coefficient −0.11, *p <* 0.001). In the pooled analysis of ASCOT, ELSA, and NPHSII, there was no association of ApoE with CVD events; the odds ratio (OR) for CVD events per 1-standard-deviation higher ApoE concentration was 1.02 (95% CI 0.96, 1.09). After adjustment for cardiovascular risk factors, the OR for CVD per 1-standard-deviation higher ApoE concentration was 0.97 (95% CI 0.82, 1.15). Limitations of these analyses include a polyclonal method of ApoE measurement, rather than isoform-specific measurement, a moderate sample size (although larger than any other study to our knowledge and with a long lag between ApoE measures), and CVD events that may attenuate an effect.

**Conclusions:**

In the largest study to date on this question, we found no evidence of an association of circulating ApoE concentration with CVD events. The established association of *APOE* genotype with CVD events may be explained by isoform-specific functions as well as other mechanisms, rather than circulating concentrations of ApoE.

## Abstract 2

Using longitudinal data, Reecha Sofat and colleagues probe the association between blood levels of ApoE and risk of cardiovascular disease.

## Author Summary

**Why Was This Study Done?:**

- Heart attacks and strokes, jointly referred to as cardiovascular disease (CVD), are the most common causes of death worldwide. Identifying risk factors that can be used to predict these events can be valuable for prevention, and identifying molecular pathways involved in risk can be useful for the development of preventive drugs.
- ApoE is a circulating protein that binds to and may regulate circulating lipoproteins, which are proteins that combine with and transport lipids and fat in the bloodstream. ApoE has several genetic variants in the human population; in previous studies, certain ApoE variants (genotypes) have been shown to be associated with greater risk of CVD.
- One hypothesis is that the amount of ApoE protein circulating in the body, which has been shown to vary by ApoE genotype, mediates the risk of CVD.

**What Did the Researchers Do and Find?:**

- We measured ApoE concentrations directly in ~10,000 individuals, ~1,400 of whom had CVD events. We did not find any association of circulating ApoE with CVD, even after adjusting for other CVD-related factors that might obscure an association.
- We also placed ApoE concentration in the context of clinical risk equations that are used to assess risk in patients, and found that it did not improve risk prediction.

**What Do These Findings Mean?:**

- This study demonstrates that ApoE genotypes that confer risk of CVD might do so via protein function rather than protein concentration in the blood.
- ApoE may still be a drug target for prevention of CVD.

## Introduction

Apolipoprotein E (ApoE) is a 34-kDa liver-derived multifunctional protein found associated with triglyceride-rich chylomicrons and very low density lipoproteins (VLDLs), their remnants, and a subset of high-density lipoprotein particles [1,2]. One of ApoE’s major physiological roles is in lipid metabolism; ApoE mediates high-affinity binding of ApoE-containing lipoproteins to the low-density lipoprotein receptor (LDL-R) and LDL receptor related protein 1 (LRP1), facilitating clearance of triglyceride-rich lipoproteins from the circulation. It is this mechanism that is thought to confer protection from atherogenesis. ApoE is a polymorphic protein with three major circulating isoforms, E2, E3, and E4 [3], and lipid metabolism is isoform dependent. Isoforms are determined by a combination of two common non-synonymous single nucleotide polymorphisms (SNPs rs7412 and rs429358) in exon 4 of the *APOE* gene on Chromosome 19 [4]. The most commonly observed, and the reference, isoform is E3. E3 has a cysteine residue at 112 and an arginine residue at 158, and is present in ~79% of the population. E4 (rs429358), the next most commonly encountered isoform (~14%), has an arginine residue substituting cysteine at 112. Finally, E2 (rs7412) is present at a frequency of ~7% and has a cysteine substituting arginine at residue 158. The resultant six common genotypes, in order of observed frequency, are ε3/ε3, ε3/ε4, ε2/ε3 ε2/ε4, ε4/ε4, and ε2/ε2. The most common genotype group is ε3/ε3, which serves as the reference category.

Very low circulating concentration of ApoE in humans is associated with early onset atherosclerosis [5]. Mice in which the *APOE* gene is deleted are prone to developing atherosclerosis [6]. Together, these findings have contributed to the view that circulating ApoE may have anti-atherogenic properties. Large population-based genetic studies in humans support this view. Carriage of ε2 is associated with higher circulating concentrations of ApoE [7], lower circulating concentrations of low-density lipoprotein cholesterol (LDL-C) [8], and a lower risk of cardiovascular disease (CVD) events [8]. In contrast, carriage of ε4 is associated with directionally opposite changes in these markers and a higher risk of CVD. It is therefore plausible that circulating concentrations of ApoE are causally associated with CVD, and, as such, ApoE could be a useful biological marker of atherosclerotic risk or a potential drug target.

To date, few studies have reported on the association of circulating ApoE concentration with CVD events (i.e., stroke and coronary heart disease [CHD]). In one study, higher circulating concentrations of ApoE were associated with a higher risk of incident CVD [9]; this contrasts with the inverse association that might be expected from animal and prior human studies. In the same population setting, a related study with a focus on stroke also found an increase in ApoE level associated with an increased risk of stroke [10]. A third study, the largest (total *n* = 2,951), also demonstrated an increase in CVD with ApoE level; however, this increase was limited to a subgroup of women with high high-density lipoprotein cholesterol (HDL-C) levels [11]. These findings require replication for a number of reasons. First, two of these studies were focussed, by design, on those aged 85 y and above. Results may not apply in younger individuals, particularly as there is evidence of an age-dependent decline in the prevalence of individuals with the ε4/ε4 genotype [12], who also have lower circulating concentrations of ApoE. Second, each study was small, with few CVD outcomes (68, 54 [strokes in stroke only study], and 156 in the three studies, respectively). Third, the ApoE-CVD associations found in these studies were adjusted for varying risk factors, but the adjustments were not uniform throughout. Lastly, initial reports of any association often yield an inflated effect estimate. This phenomenon, known as the Proteus effect or winner’s curse [13], prompts the need for replication. We therefore aimed to examine the association of ApoE concentration with CVD events in three new studies of ~10,000 middle-aged individuals in total, with a wider range of measures of cardiovascular risk factors and 1,413 CVD events.

## Methods

### Studies

Study details are described in detail elsewhere, but in brief; the Northwick Park Heart Study II (NPHSII) [14] is a prospective study that recruited 3,012 men from nine general practices across the UK with 15 y follow-up. All participants were free of CVD at the time of recruitment. Median follow-up from time of ApoE measurement was 9.9 y. There were 205 CHD events, including 136 acute myocardial infarctions (MIs), of which 50 were fatal, 65 involved coronary revascularisations, and four were silent MIs. There were 79 strokes (35 fatal). Sixteen individuals had both CHD and stroke events. In total there were 268 CVD events.

The English Longitudinal Study of Ageing (ELSA) [15] is a prospective study of household participants aged 50 y and over and resident in England. ELSA participants were recruited from respondents of the annual Health Survey for England in 1998, 1999, and 2001. In ELSA, ApoE measurement was in wave 2 (2004–2005), and participants were asked if a doctor had diagnosed MI and stroke at waves 3 (2006–2007), 4 (2008–2009), and 5 (2010–2011), a mean of 5.4 y from wave 2. Mortality and follow-up was available through the National Health Service Central Registry until 31 January 2010. Registration of death within 5 d is a legal requirement in England, so participants not registered can be assumed to be alive. Death certificates were coded using the tenth revision of the International Classification of Disease, and those categorised as CVD were extracted. During this period there were 165 MIs, of which 65 were fatal; 170 strokes, of which 40 were fatal; and 12 individuals with both diagnoses. In total, there were 323 CVD events.

The Anglo-Scandinavian Cardiac Outcomes Trial (ASCOT) [16] is a randomised clinical trial of blood-pressure- and lipid-lowering treatment in the prevention of CVD in individuals at high cardiovascular risk. A 1:1 nested case-control sample of 1,666 individuals matched for age, sex, and country of origin was used for this study. The primary endpoint of the study was combined non-fatal MI (including silent MI) and fatal CHD. Secondary endpoints included all-cause mortality, total stroke, all coronary events, total cardiovascular events and procedures, cardiovascular mortality, and non-fatal and fatal heart failure. Here, only CHD cases were included, where ascertainment was at clinical follow-up. Certified causes of death were sought, and, when available, national registries were used to find information on patients who did not return for their final visits. Endpoints were submitted and adjudicated by the ASCOT endpoints committee, the members of which were unaware of treatment assignment.

The analyses in all studies were in accordance with approval from relevant research ethics committee, and all participants provided written informed consent. See STROBE statement (S1 Text).

### ApoE Measurement

Circulating ApoE was measured using a nephelometric method on a BN II nephelometer (Siemens), using a non-isoform-specific polyclonal antibody. ApoE measures from NPHSII were made in citrated plasma taken at the fourth annual visit and were taken after a light breakfast. ELSA and ASCOT measures were made in serum. Samples used in ELSA were taken at wave 2. Most participants were fasted; those over age of 70 y (35%) and those with diabetes (7.2%) were not asked to fast (total 35.8% of ELSA participants), although most were seen prior to eating breakfast. In ASCOT, samples were taken at randomisation in the fasted state. Samples used for all measures were taken at a time prior to any CVD events. Differences in concentration dependent on whether plasma or serum was used for ApoE measurement were overcome at the analysis stage by standardising measures of ApoE.

### APOE Genotyping

The ELSA and NPHSII studies provided information on the *APOE* SNPs rs7412 and rs429358, which were used to reconstruct the ε2/ε2, ε2/ε3 ε2/ε4, ε3/ε3, ε3/ε4, and ε4/ε4 haplotypes. Genotyping in NPHSII was carried out using Taqman (Applied Biosciences), and genotyping in ELSA was based on KASP chemistry at LGC Genomics.

### Definition of Cardiovascular Events

CHD events were defined as fatal or non-fatal MI, angiographic evidence of coronary atherosclerosis, or CHD events that required intervention or where individuals underwent coronary artery bypass grafting. Stroke was defined as fatal or non-fatal; the definition encompassed both ischaemic and haemorrhagic stroke. Total CVD was a composite of CHD and stroke cases and was defined as fatal or non-fatal. Specific definitions are given in primary study reports. Comparison between definitions was carried out in order to ensure that these could be pooled for meta-analysis.

### Statistical Analysis

Statistical analysis was carried out according to a prespecified analysis plan (S2 Text). Non-normally distributed continuous variables were logarithmically transformed. For these variables we report geometric means with approximate standard deviations (SDs). Differences between case-control groups were tested by unpaired *t*-test or ANOVA, and differences in the distributions of categorical variables were assessed by the χ^2^ test. Patients with missing data were not included in the analysis. ApoE was in addition standardised, that is, rescaled to have a mean of 0 and SD of 1, to overcome any difference between sample types (e.g., serum versus plasma) across studies.

#### Association of ApoE with other circulating markers

Bivariate associations of ApoE with markers measured in each study were evaluated using Pearson correlation coefficients. Partial correlations were obtained after adjusting for age and gender. In order to investigate the shape of associations of ApoE with other circulating markers, the geometric mean of log-transformed ApoE (with 95% CI) for each decile of the associated marker was calculated and plotted separately for males and females. Only the ELSA data were used to derive these measures, as information on the widest range of measures was available from this study in both males and females, and these data are most representative of the shape of associations in the general population. NPHSII included only men, who are at higher risk of CVD than women, and ASCOT by design recruited individuals at high risk of CVD. Inclusion of both of these studies may inflate any associations of CVD biomarkers with circulating concentrations of ApoE. Reporting is therefore restricted to the most representative group.

#### Association of circulating ApoE and cardiovascular events

Multiple logistic Cox regression and Kaplan-Meier approaches were used to assess the association of ApoE concentration and CVD events. The primary outcome was total CVD, and secondary outcomes were fatal, non-fatal, and total CHD; fatal, non-fatal, and total stroke; and fatal and non-fatal CVD. Odds ratios (ORs) for all outcomes (fatal, non-fatal, and total CHD; fatal, non-fatal, and total stroke; fatal, non-fatal, and total CVD) and their corresponding 95% CIs were calculated using multiple conditional logistic regression models in ASCOT, accounting for the matching variables of age, sex, and country of origin and the treatment group to which individuals were randomised. Interaction between ApoE and randomised group was checked. Hazard ratios (HRs) with corresponding 95% CIs were calculated in the prospective studies using Cox regression models. ORs and HRs were reported in two ways: first, by SD change in ApoE concentration and, second, by tertile of ApoE concentration. Unadjusted ORs and HRs were calculated first (model 1), followed by values adjusted for total cholesterol, HDL-C, smoking, systolic blood pressure, type 2 diabetes, age, and sex (i.e., Framingham risk variables; model 2) An additional model adjusting for LDL-C was performed (model 3), given the observed genetic association of *APOE* with circulating LDL-C. For NPHSII, estimates were additionally adjusted by recruitment centre. Data from all studies were then pooled using random effects meta-analysis, weighting the effect size in individual studies by the inverse of the variance and assessing heterogeneity using the DerSimonian and Laird *Q* test, quantified using the *I* ^2^ statistic. Where time-to-event data were available, Kaplan-Meier analyses were carried out, stratifying by concentration of circulating ApoE in individuals with the baseline ε3ε3 genotype. These analyses were restricted to NPHSII. All data analysis was carried out using Stata version 12 (StataCorp).

#### Association of *APOE* genotypes with cardiovascular disease and circulating lipid markers

Haplotype associations of *APOE* were carried out using the common ε3/ε3 as the reference genotype. The association of *APOE* with circulating ApoE and other lipids was examined and the association of *APOE* with CVD was explored using Cox or logistic regression models.

## Results

Studies contributed to the analyses in different ways, summarised in S1 Table.

### Baseline Study Population Characteristics

A total of 9,587 individuals (1,642 from ASCOT, 5,389 from ELSA, and 2,556 from NPHSII) for whom measures of ApoE were available were included from all three studies. Baseline characteristics are shown in Table 1. Variables that were log_e_ transformed included ApoE, triglycerides, ferritin, fibrinogen, C-reactive protein (CRP), glucose, and body mass index. Of the 9,587 individuals with ApoE measures included in the study, 1,152 had missing data on one or more of the Framingham variables included in model 2. The proportion of missing data was 11.8% for ELSA, 1.6% for ASCOT, and 19.2% for NPHSII.

**Table 1:** Baseline characteristics of studies included.

Characteristic	NPHSII (*n =* 2,556)	ASCOT (*n =* 1,642)	ELSA (*n =* 5,389)
Age (y)	59.2 (3.3)	63.2 (8.3)	65.5 (9.3)
Sex (male)	100%	85.9%	44.4%
Current smokers	27.4%	33.5%	14.1%
Systolic blood pressure (mm Hg)	133.4 (18.1)	164.7 (17.7)	135.2 (18.7)
Diastolic blood pressure (mm Hg)	81.8 (10.8)	95.2 (10.2)	75.7 (10.9)
Total cholesterol (mmol/l)	5.63 (0.97)	5.94 (1.05)	6.00 (1.17)
High-density lipoprotein cholesterol (mmol/l)	1.72 (0.59)	1.26 (0.34)	1.54 (0.39)
Low-density lipoprotein cholesterol (mmol/l)	3.08 (1.01)	3.85 (0.93)	3.66 (0.97)
Triglycerides (mmol/l)*	1.81 (0.93)	1.65 (0.80)	1.57 (0.81) (*n =* 5,386)
Apolipoprotein E (mg/l)*	34.9 (11.0)	49.4 (15.4)	39.4 (12.1)
Apolipoprotein A (g/l)	1.64 (0.32)	—	—
Apolipoprotein B (g/l)	0.86 (0.24)	—	—
Lipoprotein(a) (mg/dl)	8.1 (11.6)	—	—
Diabetes	2.1%	26.0%	7.2%
Glucose (mmol/l)*	—	6.05 (1.62) (*n =* 1,480)	4.95 (0.72)
Body mass index (kg/m^2^)*	26.4 (3.5)	28.4 (4.0)	27.4 (4.5)
Waist-to-hip ratio	—	—	0.89 (0.08)
C-reactive protein (mg/dl)*	2.38 (2.44)	—	2.02 (2.24)
Haemoglobin (g/dl)	—	—	14.3 (1.4)
Ferritin (ng/ml)*	—	—	86.9 (72.4)
Fibrinogen (g/l)*	2.88 (0.56)	—	3.13 (0.68)

Data are presented as mean (SD) or percent. Dashes indicate missing variables in the given dataset.

*Log transformed; geometric means and approximate SDs are presented.

ASCOT, Anglo-Scandinavian Cardiac Outcomes Trial; ELSA, English Longitudinal Study of Ageing; NPHSII, Northwick Park Heart Study II; SD, standard deviation.

### Cross-Sectional Associations of ApoE with Cardiovascular Risk Factors and Other Variables

**Fig 1:**
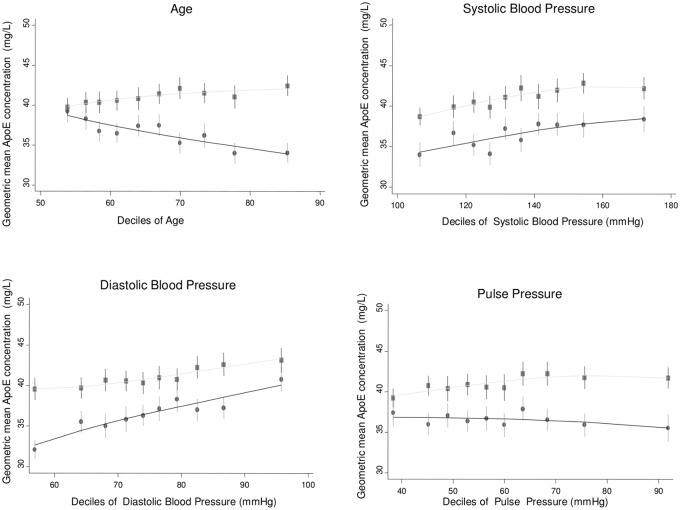
Cross-sectional association between geometric mean of ApoE concentration and age, systolic blood pressure, diastolic blood pressure, and pulse pressure measured in ELSA, by gender. Male (circles) and female (square) mean log ApoE concentration was calculated for each decile of the associated variable and plotted to visualise the shape of the association. Bars give 95% CIs.

**Fig 2:**
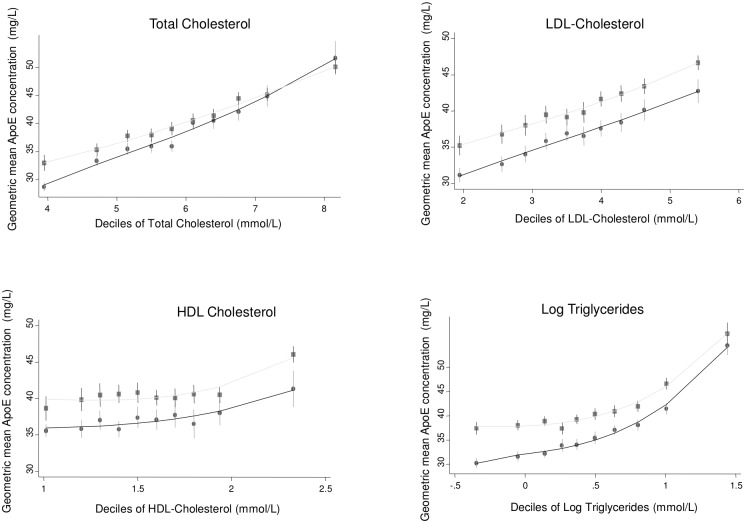
Cross-sectional association between geometric mean of ApoE concentration and total cholesterol, low-density lipoprotein cholesterol, high-density lipoprotein cholesterol, and triglycerides measured in ELSA, by gender. Male (circles) and female (square) mean log ApoE concentration was calculated for each decile of the associated variable and plotted to visualise the shape of the association. Bars give 95% CIs.

**Fig 3:**
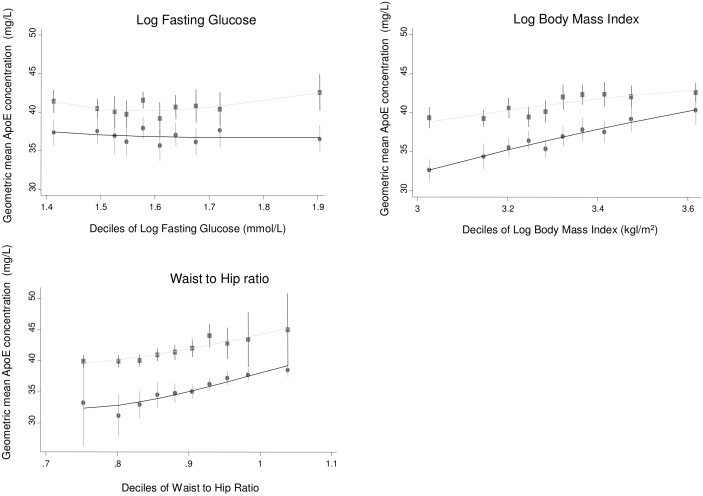
Cross-sectional association between geometric mean of ApoE concentration and glucose, body mass index, and waist-to-hip ratio measured in ELSA, by gender. Male (circles) and female (square) mean log ApoE concentration was calculated for each decile of the associated variable and plotted to visualise the shape of the association. Bars give 95% CIs.

**Fig 4:**
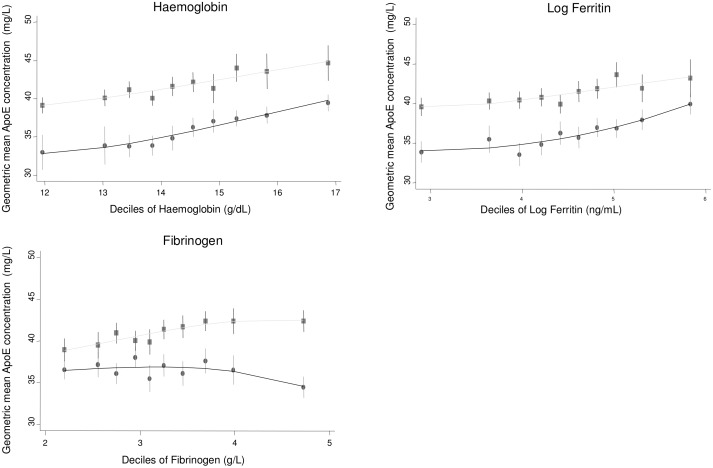
Cross-sectional association between geometric mean of ApoE concentration and haemoglobin, ferritin, and fibrinogen measured in ELSA, by gender. Male (circles) and female (square) mean log ApoE concentration was calculated for each decile of the associated variable and plotted to visualise the shape of the association. Bars give 95% CIs.

**Fig 5:**
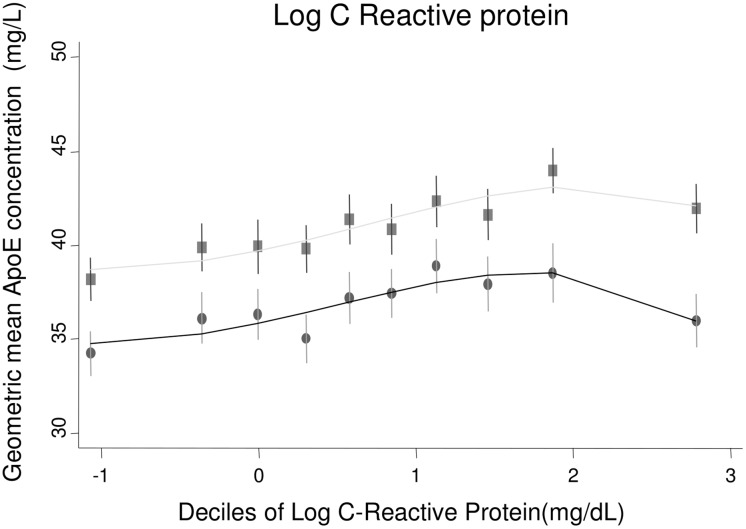
Cross-sectional association between geometric mean of ApoE concentration and C-reactive protein measured in ELSA, by gender. Male (circles) and female (square) mean log ApoE concentration was calculated for each decile of the associated variable and plotted to visualise the shape of the association. Bars give 95% CIs.

Bivariate associations of log-transformed ApoE with other risk factors were assessed in the two population-based studies ELSA and NPHSII (Table 2). Significant associations were observed with a number of cardiovascular risk factors, including systolic and diastolic blood pressure; total cholesterol, LDL-C, HDL-C, and triglycerides; and body mass index (Figs 1–3; Table 2). ApoE was also significantly associated with ApoB (correlation coefficient 0.174, *p <* 0.001) and lipoprotein(a) (correlation coefficient −0.12, *p <* 0.001) in NPHSII, where these measures were available, but not with ApoA1 (correlation coefficient 0.02, *p =* 0.46). In addition, circulating ApoE was significantly associated with markers of inflammation including CRP (measured in ELSA and NPHSII, correlation coefficient 0.11 and 0.13, respectively, *p <* 0.001 for both), ferritin (ELSA only, correlation coefficient 0.07, *p <* 0.001), and, to a lesser extent, fibrinogen (correlation coefficient 0.05, *p <* 0.001, in ELSA, and 0.01, *p =* 0.52, in NPHSII) (Figs 4 and 5; Table 2). There were no significant differences following adjustment for age or gender.

**Table 2:** Bivariate associations of circulating ApoE with measured markers in the population-based studies, ELSA and NPHSII.

Marker	ELSA		NPHSII	
	Correlation Coefficient	*p-*Value	Correlation Coefficient	*p-*Value
**Blood pressure**				
Systolic blood pressure (mm Hg)	0.08	<0.001	0.08	<0.001
Diastolic blood pressure (mm Hg)	0.10	<0.001	0.11	<0.001
Pulse pressure (mm Hg)	0.02	0.10	0.03	0.19
**Circulating lipids**				
Total cholesterol (mmol/l)	0.46	<0.001	0.34	<0.001
High-density lipoprotein cholesterol (mmol/l)	0.16	<0.001	−0.14	<0.001
Low-density lipoprotein cholesterol (mmol/l)	0.30	<0.001	0.14	<0.001
Triglycerides (mmol/l)*	0.43	<0.001	0.46	<0.001
Apolipoprotein B (g/l)	NA	NA	0.13	<0.001
Apolipoprotein A1 (g/l)	NA	NA	0.01	0.64
Lipoprotein(a) (mg/dl)	NA	NA	−0.11	<0.001
**Haemotological markers**				
Haemoglobin (g/dl)	0.04	<0.001	NA	NA
Ferritin (ng/ml)*	0.07	<0.001	NA	NA
Fibrinogen (g/l)*	0.05	<0.001	0.01	0.52
**Inflammatory markers**				
C-reactive protein (mg/l)*	0.11	<0.001	0.13	<0.001
**Glucose and anthropometric measures**				
Fasting glucose (mmol/l)*	−0.00	0.87	NA	NA
Body mass index (kg/m^2^)*	0.12	<0.001	0.19	<0.001
Waist-to-hip ratio	−0.02	0.18	NA	NA
**Lifestyle measures**				
Current smoking	0.03	0.01	−0.10	0.65

No differences were observed when analyses were further adjusted for age and gender.

*Log transformed.

ELSA, English Longitudinal Study of Ageing; NA, not available; NPHSII, Northwick Park Heart Study II.

### Association of ApoE Concentrations and Cardiovascular Disease during Follow-Up

**Fig 6:**
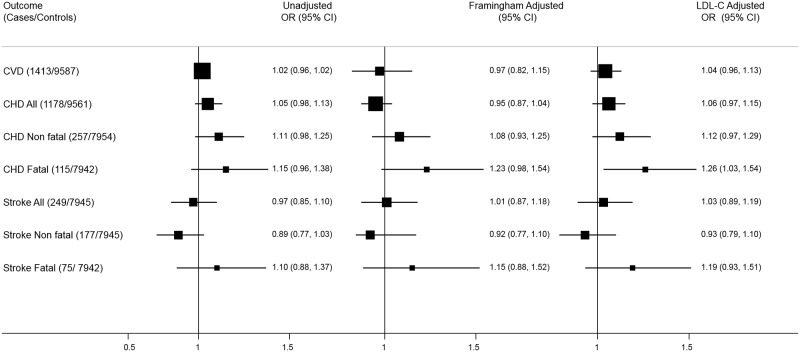
Meta-analysis of association of ApoE level with coronary heart disease and stroke. Adjusted (for Framingham variables) and unadjusted summaries are given separately. ORs represent per SD change of ApoE. Numbers of events and total numbers included in each analysis are given in S4 Table. CHD, coronary heart disease; CVD, cardiovascular disease; LDL-C, low-density lipoprotein cholesterol; OR, odds ratio; SD, standard deviation.

There were no statistically significant differences in circulating concentrations of ApoE between CVD cases and controls for all three studies (Table 3). There was no association of ApoE concentration either per SD increase or by tertile with CVD, with no additional evidence of a trend of association across tertiles (Table 4). There was no significant association between ApoE concentration and CVD in individual studies (Table 4) or when estimates were pooled using random effects meta-analysis (OR 1.02, 95% CI 0.96, 1.09) or after adjustment (Framingham-adjusted OR 0.97, 95% CI 0.82, 1.15) (Fig 6). Adjustment for LDL-C alone also did not impact the overall association (OR 1.04, 95% CI 0.96, 1.13). When CHD (all CHD, fatal and non-fatal) and stroke (all stroke, fatal and non-fatal) endpoints were assessed separately across all three studies, there was no significant association of circulating concentration of ApoE with either outcome (Fig 6; S2 Table). For the crude associations, analysis of ApoE quintiles and CVD risk was carried out and a quadratic model was fitted following peer review to confirm the absence of a U-shaped association as an explanation of a null effect (S3 Table).

**Table 3:** ApoE concentrations in individuals who later developed cardiovascular disease by case/control status in all three studies.

Study	Geometric Mean of ApoE Concentration (mg/l) (Approximate Standard Deviation) [*n*]		*p-*Value
	Control	Case	
ASCOT	49.01 (15.67) [820]	49.83 (15.12) [822]	0.28
ELSA	39.34 (12.03) [5,066]	39.47 (12.71) [323]	0.85
NPHSII	34.82 (10.98) [2,288]	35.98 (10.68) [268]	0.11

**Table 4:** Summary estimates for the association of log ApoE concentration with cardiovascular events from the three studies.

ApoE Concentration	Model 1		Model 2		Model 3	
	OR or HR (95% CI)	*p-*Value	OR or HR (95% CI)	*p-*Value	OR or HR (95% CI)	*p-*Value
**ASCOT**						
Per SD increase in log ApoE	0.99 (0.89, 1.10)	0.89	0.83 (0.74, 0.94)	0.003	0.96 (0.85, 1.08)	0.52
Tertile 1	Ref		Ref		Ref	
Tertile 2	1.07 (0.84, 1.36)	0.58	0.89 (0.69, 1.15)	0.05	1.23 (0.93, 1.62)	0.79
Tertile 3	1.07 (0.83, 1.39)		0.74 (0.56, 1.00)		1.03 (0.77, 1.38)	
**ELSA**						
Per SD increase in log ApoE	1.01 (0.90, 1.13)	0.85	1.08 (0.94, 1.25)	0.27	1.08 (0.95, 1.22)	0.23
Tertile 1	Ref		Ref		Ref	
Tertile 2	0.97 (0.74, 1.29)	0.65	1.04 (0.75, 1.44)	0.32	1.11 (0.84, 1.48)	0.12
Tertile 3	1.07 (0.81, 1.39)		1.19 (0.85, 1.66)		1.25 (0.94, 1.66)	
**NPHSII**						
Per SD increase in log ApoE	1.08 (0.96, 1.22)	0.20	1.03 (0.90, 1.19)	0.66	1.09 (0.95, 1.25)	0.21
Tertile 1	Ref		Ref		Ref	
Tertile 2	1.24 (0.92, 1.68)	0.23	1.53 (1.07, 2.17)	0.51	1.41 (1.00, 1.99)	0.16
Tertile 3	1.21 (0.89, 1.63)		1.16 (0.81, 1.68)		1.30 (0.92, 1.84)	

Estimates are presented as the odds of cardiovascular disease per SD increase in ApoE concentration and as the OR per tertile of ApoE, where the reference group is tertile 1. *p-*Values represent a test of trend across ApoE tertiles. Model 1 is unadjusted; model 2 is Framingham adjusted; model 3 is LDL-C adjusted; ORs are reported for the ASCOT case-control subset and ELSA, and HRs are reported for the prospective NPHSII.

ASCOT, Anglo-Scandinavian Cardiac Outcomes Trial; ELSA, English Longitudinal Study of Ageing; HR, hazard ratio; NPHSII, Northwick Park Heart Study II; OR, odds ratio; SD, standard deviation.

**Fig 7:**
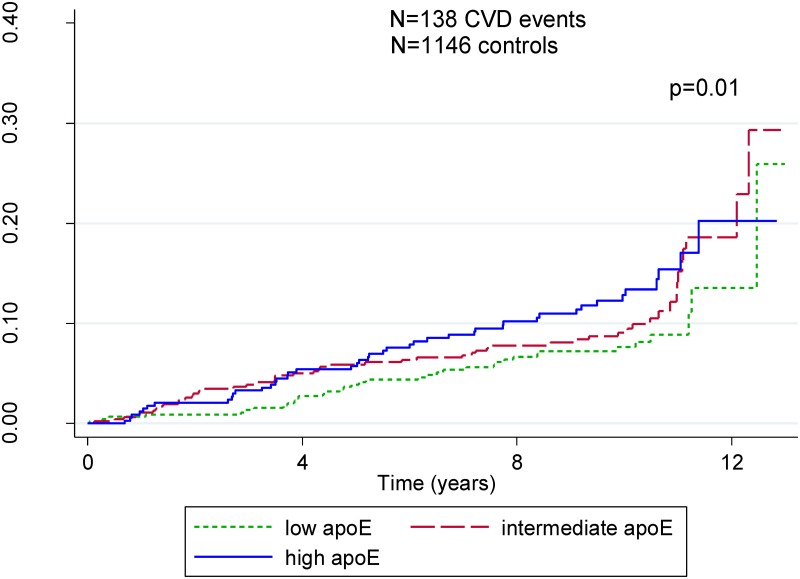
Kaplan-Meier plot for tertiles of ApoE concentration in individuals with ε3ε3 genotype. This analysis was conducted to overcome any effect of isoform or structural change in the circulating protein. High concentrations are indicated in blue, intermediate in red, and low in green. Precise time-to-event measures are available in NPHSII, and therefore analyses are restricted to this study. There is a marginally statistically significant relationship between ApoE concentration and time to CVD event; however, given that this analysis is restricted to one study, which included only men, it has to be interpreted with caution. CVD, cardiovascular disease.

In an exploratory analysis restricted to the sub-sample of individuals from NPHSII with the ε3ε3 genotype, we conducted a time-to-event analysis by tertile of ApoE concentration to overcome any isoform-specific effect. The Kaplan-Meier curves showed no major differences in incidence of CVD events according to ApoE concentration (Fig 7).

### Association of *APOE* Genotypes with Cardiovascular Disease and Circulating Lipid Markers

Levels of circulating lipids—including total, LDL-, and HDL-C and triglycerides—and ApoE in the *APOE* genotype groups were as predicted from previous studies (S4 Table). CVD events have previously been shown to be associated with *APOE* genotype. Genotype was available in NPHSII and ELSA, and this genotypic effect is replicated on a smaller scale (S5 Table).

## Discussion

### Summary of Main Findings

We have described the molecular epidemiology of circulating ApoE in a large study of middle-aged individuals that was population-based, in contrast to previous studies, which have focused on ApoE concentrations in related individuals [7]. We have confirmed here that circulating ApoE concentration is associated with the levels of major circulating lipids and lipoproteins including total cholesterol, LDL-C, and HDL-C as well as triglycerides; this finding is consistent with published findings [7]. We further investigated the association of circulating ApoE concentration and CVD risk in ~10,000 individuals including 1,413 CVD cases. In contrast to prior reports [9–11], we found no association between ApoE concentration and CVD events, either when expressed as an OR per SD change of ApoE concentration or when examined by tertile of ApoE concentration or by subtype of CVD.

### Discrepancy with Previously Published Studies

The lack of an association between circulating ApoE and CVD contrasts with other studies examining this association [9–11] and with an anticipated CVD protective effect of higher circulating ApoE. In explaining the discrepancy of findings between the current and previous reports, the following points are important. First, the study presented here includes individuals who are middle-aged and are more likely to represent the major group targeted for primary CVD prevention. Two of the previous reports included only individuals ≥85 y old, a population that may display a survivor bias. Moreover, it is known that the relative frequency of the ε4ε4 genotype group, which is strongly associated with lower circulating ApoE concentrations, declines with age [12]. Therefore, older populations are predicted to have higher circulating ApoE concentration (associated with enrichment for the ε2 and ε3 alleles). We did observe an interaction between age and sex (ELSA: *p* for interaction < 0.001; ASCOT: *p* for interaction = 0.02). This interaction has also been observed in other studies that measured ApoE at scale [17]. This interaction could be due to genotypic effect or could be a result of the effect of other unmeasured lipid intermediates that could also alter circulating levels of ApoE in men and women. The association of ApoE with CVD risk was also replicated in PREVEND [11], a more representative sample; however, in this study a borderline association was observed between circulating ApoE and CVD risk in women only. Second, all CVD cases in the current analysis are incident events, and this should largely overcome any issues of reverse causality that can affect conclusions drawn from the retrospective case-control or cross-sectional designs of some, but not all, previous reports [9,10]. Lastly, the size of the current analysis (1,413 cases, five times more than the largest published study), and the replication of findings across three different datasets, overcomes some of the limitations of prior reports on the association of circulating ApoE and CVD. During the review process of this manuscript, a large (~92,000 individuals) population-based study examining the association of ApoE and CHD was published [18] demonstrating that circulating ApoE increases CHD risk. It is interesting to note that our findings from all CHD (OR 1.05, 95% CI 0.98, 1.13) approach their findings (OR for ischaemic heart disease of 1.15, 95% CI 1.04, 1.27; OR for MI of 1.16, 95% CI 1.00, 1.36), however do not reach significance. Moreover, the association reported by Rasmussen et al. is limited to men and attenuates with the adjustment of triglycerides. Like the other published studies, Rasmussen et al. did not demonstrate a convincing association with ApoE, despite a greater power to detect such an association. The findings that they report are also contrary to the biological prior from genetic and animal studies, that higher ApoE concentration should be protective against atherosclerotic disease.

### Placing Findings in the Context of Known Genetics of *APOE* and Biological Functions of ApoE

The observation that individuals with very low or completely absent circulating ApoE develop early atherosclerosis, and that this has a genetic basis [5], has been influential in the development of hypotheses on the role of ApoE in atherosclerosis. The largest genetic study investigating the association of *APOE* mutations with CVD support this hypothesis [8]. For the ε2ε2 (associated with higher circulating ApoE) versus ε3ε3 comparison, Bennet et al. [8] demonstrated an OR of 0.80 (95% CI 0.70, 0.90) for CVD. Our data do not reach significance; for the same comparison, we have 30% power to detect an OR of this size at a 5% significance level using 377 cases and 4,709 controls, but the trend of the effect is similar (S5 Table). Human studies have been dominated by evaluation of the relationship of *APOE* genotype with a range of diseases including CVD, Alzheimer disease [19], and age-related macular degeneration [20]. Together with animal studies, these human studies have contributed to the prevailing view that circulating ApoE is anti-atherogenic. For example, carriage of ε2 is associated with higher circulating concentration of ApoE, lower LDL-C concentration, and lower risk of CVD [8]. Conversely, carriage of ε4 is associated with directionally opposite changes in ApoE and LDL-C concentration and a higher risk of CVD. These findings have been interpreted as a consequence of the functional differences between isoforms of ApoE, namely, lipid and receptor binding, rather than circulating concentrations of the protein. For example, the E2 isoform binds LDL-R and LRP1 with a reduced efficiency compared to the other isoforms (≤2% compared to E3; E4 has affinities comparable to E3) [21,22]. This reduced binding efficiency has been thought to result in up-regulation of expression of LDL-R and LRP1, with the consequent effect of increasing clearance and lowering circulating concentrations of pro-atherogenic VLDLs, chylomicrons, and their remnants. E2 as well as E3 preferentially bind to small phospholipid-rich HDL-C particles, in contrast to E4, which binds larger pro-atherogenic triglyceride-rich particles [23]. Binding with HDL-C supports an additional role of E2 in reverse cholesterol transport, as ApoE acts as a ligand for high-density-lipoprotein-mediated cholesterol delivery to the liver. However, the present study indicates a null association of ApoE concentration with CVD risk overall and in analyses restricted to individuals with the ε3/ε3 genotype.

One of the functional consequences of the reduced binding to LDL-R and LRP1 of E2 could be higher detectable concentrations of ApoE. This has been interpreted as higher levels of circulating ApoE being protective. However, a higher detectable concentration of ApoE could be a paradoxical increase as a consequence of the genetic point mutation rather than being causal in itself. One way in which ApoE concentration might be useful in CVD prediction is with isoform-specific measures. The predictive utility of isoforms could then be tested in large populations. However, it should be borne in mind that, given the distribution of cases and controls amongst genotype groups, most cases will occur in those with circulating ApoE3, as these individuals make up the majority of the population (S1 Fig). Most cases of CVD will therefore have circulating concentrations of ApoE within the normal range (i.e., within 2 SDs of the population mean). However, that does not preclude ApoE being a useful target for CVD; rather, any drug would need to recapitulate the pattern of lipids seen with circulating E2. Such a drug could be useful in individuals with any genotype.

Two other courses of investigation to more precisely defining the role of ApoE in health and disease are now necessary. First, the view that *APOE* confers disease risk (and/or protection) mediated through LDL-C may be over simplistic. Functional studies of all isoforms indicate the importance of ApoE in the clearance of other potentially atherogenic lipid particles including VLDLs, chylomicrons, and their remnants. To date, studies in small samples (~200 individuals) [24] have indicated that there is indeed a differential effect of *APOE* genotype on circulating lipids other than the “traditionally” measured LDL-C, HDL-C, and triglycerides. Investigation of other lipid intermediates and their association with *APOE* now requires a population-level effort and may prove to be more fruitful than ApoE with respect to predictive utility for CVD. Measurement of these lipid intermediates is now possible in a time- and cost-efficient way using high-throughput mass spectrometry and nuclear magnetic resonance platforms. Second, the view that the genetic variation in the *APOE* locus that influences CVD risk is limited to the ε2/ε3/ε4 haplotype is also being challenged. Genetic studies using genome-wide [25] and gene-centric SNP arrays [26] implicate genes flanking *APOE*, including *BCL3*, *PVRL2*, *TOMM40*, and *APOC1-C4-C2*, rather than *APOE* itself in regulation of circulating lipid concentrations, in particular LDL-C, as well as additional variants within the *APOE* gene. The strength of these associations is greater than that with SNPs in *APOE* alone. Limitations of some of these studies include the fact that both SNPs that determine the ε2/ε 3/ε 4 haplotype (rs429358 and rs7412) are not included on all SNP arrays or are not well imputed and therefore have not been identified as signals in such analyses. However, even where these SNPs are directly typed and included in analyses, the strength of the association with circulating lipids is greater from the flanking regions [26]. Results from large genome-wide association studies and consortia data can now be used to inform further functional and molecular analyses, in order to truly assess the effect of *APOE* and circulating ApoE on disease risk. This is not only of interest in CVD, but also in other diseases of ageing including Alzheimer disease, where the risk conferred by *APOE* ε4 is >5-fold greater than that seen in CVD, and age-related macular degeneration, where, in contrast to Alzheimer disease and CVD, ε2 confers an increase in risk of disease and ε4 confers a protective effect.

### Limitations of This Study

It is important to address some limitations of this study. First, the assay used here is commercial, using a polyclonal antibody. It does not discriminate between isoforms of circulating ApoE. However, the pattern of observing higher circulating concentrations with carriage of E2 and lower concentrations with carriage of E4 is preserved and consistent with other studies. We are therefore confident that the concentrations measured are a reflection of the underlying genotype. Second, although the sample size is larger than that of previously reported studies, compared to studies that reliably estimate the risk of a marker for a disease, it is still moderate. These studies require further replication in order to more precisely delineate the direction and magnitude of the effect. Third, we are unable to differentiate between ischaemic and haemorrhagic stroke in the presented results, but, given that haemorrhagic stroke represents 10%–15% of stroke cases in the general population (https://www.strokeaudit.org) and a total of 249 strokes are included, it is doubtful that this would bias the results. Finally, samples that were used for measurement were taken before incident events, and the median time before an event across all included studies was 5.5 y. This time between sampling and event could be one explanation of an attenuated effect. However, the follow-up time is comparable to that of other published studies that do show a relationship of circulating ApoE with CVD, even with small sample sizes. One way in which this variability of ApoE could be overcome is to construct and then adjust for regression dilution ratios. Ratios are based on regression of serial measures of ApoE on baseline levels with any additional and necessary adjustments. However, serial measures were available neither in the studies presented here nor in the current ApoE literature, and these analyses will be possible only once these data emerge. Exploratory analysis applying to ApoE regression dilution ratios calculated by the CRP-CHD Genetics Consortium for another blood-based biomarker, CRP (where the regression dilution ratio adjusted for age and sex was 0.57, 95% CI 0.51, 0.64) [27], indicated that the effect of regression to the mean is unlikely to have a major effect on the observed association, with the odds of CVD for a 1-SD increase in ApoE increasing from 1.02 to 1.035. These analyses were carried out following editorial comments during the review process.

In order to more precisely link genetic variation in the *APOE* gene locus to disease and biomarkers, finer resolution of the genetic variation in this region is required. This can be achieved through next-generation sequencing or imputation against broad (1000 Genomes) and deep marker panels. Integrating this information with circulating concentrations of ApoE and a range of other biomarkers, and specifically lipid intermediates, may clarify the link between genotype, circulating ApoE, and CVD and other diseases. This will inform the clinical utility of tests based on measurement of either genotype or circulating ApoE, or of indeed targeting ApoE with new therapeutic agents.

## Supporting Information

S1 Fig Distribution of coronary heart disease cases and controls stratified by *APOE* haplotype. Adapted from Bennet et al. [8]. The ε3ε3 genotype accounts for the majority of the population and also accounts for average levels of ApoE. The putatively protective ε2ε2 genotype, associated with lower levels of ApoE and LDL-C, accounts for fewer individuals, as does ε4ε4, associated with higher risk of CVD and higher circulating ApoE. This distribution of genotypes may be one explanation for the lack of an association between circulating ApoE and CVD.
(DOCX)

S1 Table Contribution of studies to analyses presented. (DOCX)

S2 Table Numbers of events and total numbers of individuals that contributed to the analyses shown in Fig 6. (DOCX)

S3 Table Analysis of cardiovascular disease outcomes and ApoE by quintiles and quadratic model, to demonstrate the absence of a U-shaped effect as an explanation for the null result. Analyses were carried out only on the crude (unadjusted) model.
(DOCX)

S4 Table Association of circulating lipids and ApoE with *APOE* genotype. (DOCX)

S5 Table Cardiovascular disease risk according to *APOE* genotype. Analysis is restricted to ELSA and NPHSII, for which genotype data are available. Genotype distributions conformed to Hardy-Weinberg expectations, and allele frequencies were not significantly different from those reported in previous studies of individuals in the UK.
(DOCX)

S1 Text STROBE statement. (DOC)

S2 Text Analysis plan. (DOC)

## Abbreviations

ASCOT: Anglo-Scandinavian Cardiac Outcomes Trial
CHD: coronary heart disease
CRP: C-reactive protein
CVD: cardiovascular disease
ELSA: English Longitudinal Study of Ageing
HDL-C: high-density lipoprotein cholesterol
HR: hazard ratio
LDL-C: low-density lipoprotein cholesterol
MI: myocardial infarction
NPHSII: Northwick Park Heart Study II
OR: odds ratio
SD: standard deviation
VLDL: very low density lipoprotein
