# Supplementary material for: Circulating Apolipoprotein E Concentration and Cardiovascular Disease Risk: Meta-analysis of Results from Three Studies
Source: PLoS Med. 2016 Oct 18;13(10):e1002146. doi: 10.1371/journal.pmed.1002146 (PMC5068709; doi:10.1371/journal.pmed.1002146)
Supplement: S1 Fig — Adapted from Bennet et al. [8]. The ε3ε3 genotype accounts for the majority of the population and also accounts for average levels of ApoE. The putatively protective ε2ε2 genotype, associated with lower levels of ApoE and LDL-C, accounts for fewer individuals, as does ε4ε4, associated with higher risk of CVD and higher circulating ApoE. This distribution of genotypes may be one explanation for the lack of an association between circulating ApoE and CVD. (DOCX) [file pmed.1002146.s001.docx]

**S1 Figure**  Distribution of CHD cases and controls stratified by APOE haplotype. Adapted from Bennet et al. ([8](#_ENREF_8)). The ε3ε3 genotype accounts for the majority of the population and also accounts for average levels of ApoE. The putative protective ε2ε2 genotype, associated with lower levels of ApoE and LDL-C also account for fewer individuals as does ε4ε4, associated with higher risk of CVD and higher circulating ApoE. This may be one explanation of a lack of an association between circulating ApoE and CVD.
