# Supplementary material for: Circulating Apolipoprotein E Concentration and Cardiovascular Disease Risk: Meta-analysis of Results from Three Studies
Source: PLoS Med. 2016 Oct 18;13(10):e1002146. doi: 10.1371/journal.pmed.1002146 (PMC5068709; doi:10.1371/journal.pmed.1002146)
Supplement: S1 Table — (DOCX) [file pmed.1002146.s002.docx]

S1Table Contribution of studies to analyses presented

| Study | CHD | Stroke | ApoE-Biomarker Associations | Kaplan Meier |
| --- | --- | --- | --- | --- |
| ASCOT | ● |  |  |  |
| ELSA | ● | ● | ● |  |
| NPHSII | ● | ● | ● | ● |
