# Supplementary material for: Circulating Apolipoprotein E Concentration and Cardiovascular Disease Risk: Meta-analysis of Results from Three Studies
Source: PLoS Med. 2016 Oct 18;13(10):e1002146. doi: 10.1371/journal.pmed.1002146 (PMC5068709; doi:10.1371/journal.pmed.1002146)
Supplement: S2 Table — (DOCX) [file pmed.1002146.s003.docx]

**S2 Table** Numbers of events and total numbers of individuals that have contributed to analyses shown in Figure 2

|  | **Number of Events/Total N** | | |
| --- | --- | --- | --- |
| Outcome | Unadjusted | Framingham adjusted | LDL adjusted |
| CVD | 1413/9587 | 1304/8491 | 1169/8642 |
| CHD  ALL  NF  Fatal | 1178/9561  257/7945  115/7942 | 1106/8491  206/6875  94/6874 | 962/8642  223/7368  104/7365 |
| Stroke  ALL  NF  Fatal | 249/7945  177/7945  75/7942 | 222/6875  160/6875  65/6874 | 233/7368  168/7368  68/7365 |
