# Supplementary material for: Circulating Apolipoprotein E Concentration and Cardiovascular Disease Risk: Meta-analysis of Results from Three Studies
Source: PLoS Med. 2016 Oct 18;13(10):e1002146. doi: 10.1371/journal.pmed.1002146 (PMC5068709; doi:10.1371/journal.pmed.1002146)
Supplement: S3 Table — Analyses were carried out only on the crude (unadjusted) model. (DOCX) [file pmed.1002146.s004.docx]

**S3 Table** Analysis of ApoE by quintiles and quadratic model on CVD outcomes, to demonstrate the absence of a U shaped effect as an explanation for the null result. Analyses were carried out only on the crude, unadjusted model.

|  | **Model 1** | | **P value for** |
| --- | --- | --- | --- |
|  | **OR/HR (95% CI)** | **P value** | **Quadratic effect** |
| **ASCOT** |  |  |  |
| Quintile 1 | Ref |  | P=0.09 |
| Quintile 2 | 1.18 (0.85-1.65) | 0.32 |  |
| Quintile 3 | 1.13 (0.82-1.56) | 0.45 |  |
| Quintile 4 | 1.15 (0.83-1.60) | 0.40 |  |
| Quintile 5 | 1.04 (0.75-1.45) | 0.82 |  |
| **ELSA** |  |  |  |
| Quintile 1 | Ref |  | P=0.32 |
| Quintile 2 | 0.84 (0.59-1.21) | P=0.36 |  |
| Quintile 3 | 0.81 (0.58-1.15) | P=0.25 |  |
| Quintile 4 | 0.99 (0.70-1.39) | P=0.95 |  |
| Quintile 5 | 0.99 (0.71-1.39) | P=0.97 |  |
| **NPHSII** |  |  |  |
| Quintile 1 | Ref |  | P=0.34 |
| Quintile 2 | 1.30 (0.88-1.92) | 0.18 |  |
| Quintile 3 | 1.25 (0.83-1.89) | 0.28 |  |
| Quintile 4 | 1.17 (0.78-1.75) | 0.45 |  |
| Quintile 5 | 1.32 (0.89-1.96) | 0.16 |  |
