# Supplementary material for: Circulating Apolipoprotein E Concentration and Cardiovascular Disease Risk: Meta-analysis of Results from Three Studies
Source: PLoS Med. 2016 Oct 18;13(10):e1002146. doi: 10.1371/journal.pmed.1002146 (PMC5068709; doi:10.1371/journal.pmed.1002146)
Supplement: S4 Table — (DOCX) [file pmed.1002146.s005.docx]

**S4 Table** : Association of circulating lipids and ApoE with *APOE* genotype. Rank order follows that ε3ε3 genotype has the average of all the circulating lipids.

| **Variable** | **APOE haplotype** | **NPHS-II** | **ELSA** |
| --- | --- | --- | --- |
| ApoE (mg/L) | 22 | 65.4 (20.0) | 83.2 (25.5) |
|  | 23 | 43.8 (12.3) | 51.0 (12.3) |
|  | 24 | 40.0 (13.8) | 47.6 (11.7) |
|  | 33 | 33.9 (9.6) | 38.8 (10.4) |
|  | 34 | 31.7 (9.5) | 34.5 (9.7) |
|  | 44 | 28.2 (10.7) | 28.0 (11.2) |
| Total cholesterol (mmol/L) | 22 | 4.98 (0.72) | 5.28 (1.14) |
|  | 23 | 5.26 (0.98) | 5.66 (1.06) |
|  | 24 | 5.46 (1.10) | 5.76 (1.13) |
|  | 33 | 5.69 (0.96) | 6.01 (1.17) |
|  | 34 | 5.79 (0.96) | 6.15 (1.22) |
|  | 44 | 5.82 (0.88) | 6.37 (1.29) |
| HDL cholesterol (mmol/L) | 22 | 2.03 (0.47) | 1.55 (0.37) |
|  | 23 | 1.72 (0.59) | 1.55 (0.39) |
|  | 24 | 1.82 (0.67) | 1.53 (0.41) |
|  | 33 | 1.72 (0.59) | 1.55 (0.39) |
|  | 34 | 1.71 (0.62) | 1.51 (0.38) |
|  | 44 | 1.57 (0.53) | 1.56 (0.38) |
| LDL cholesterol (mmol/L) | 22 | 2.14 (0.77) | 2.76 (0.76) |
|  | 23 | 2.69 (1.09) | 3.25 (0.89) |
|  | 24 | 2.90 (1.04) | 3.39 (0.87) |
|  | 33 | 3.14 (0.98) | 3.68 (0.96) |
|  | 34 | 3.23 (1.00) | 3.84 (1.01) |
|  | 44 | 3.56 (0.92) | 3.94 (1.04) |
| Triglyceride (mmol/L) | 22 | 1.71 (0.81) | 1.89 90.96) |
|  | 23 | 1.90 (1.00) | 1.70 (0.88) |
|  | 24 | 1.69 (0.89) | 1.71 (0.89) |
|  | 33 | 1.77 (0.89) | 1.52 (0.78) |
|  | 34 | 1.83 (0.95) | 1.62 (0.83) |
|  | 44 | 1.80 (0.79) | 1.59 (0.93) |
