# Supplementary material for: Circulating Apolipoprotein E Concentration and Cardiovascular Disease Risk: Meta-analysis of Results from Three Studies
Source: PLoS Med. 2016 Oct 18;13(10):e1002146. doi: 10.1371/journal.pmed.1002146 (PMC5068709; doi:10.1371/journal.pmed.1002146)
Supplement: S5 Table — Analysis is restricted to ELSA and NPHSII, for which genotype data are available. Genotype distributions conformed to Hardy-Weinberg expectations, and allele frequencies were not significantly different from those reported in previous studies of individuals in the UK. (DOCX) [file pmed.1002146.s006.docx]

**S5 Table** CVD risk according to *APOE* genotype, analysis is restricted to ELSA and NPHS II where genotype data are available. Genotype distributions conformed to Hardy-Weinberg expectations and allele frequencies were not significantly different from that reported in previous studies of individuals in the UK.

|  | **NPHS II** | **ELSA** | **Combined studies** | **P value** |
| --- | --- | --- | --- | --- |
| *APOE* Haplotype | Unadjusted HR (95% CI) Number of events/total | Unadjusted OR (95% CI) Number of events/total | Unadjusted HR (95% CI) |  |
| ε2 ε 2 | NA  0/16 | 1.11 (0.13-4.46)  2/29 | 1.11 (0.13-4.46)  2/29 | 1.00 |
| ε 2 ε 3 | 0.70  (0.46-1.06) 26/345 | 0.92 (0.63-1.33)  35/604 | 0.82 (0.62-1.08)  61/949 | 0.15 |
| ε 2 ε 4 | 1.37  (0.56-3.34) 5/41 | 1.85 (1.02-3.35)  13/118 | 1.69 (1.03-2.77)  18/159 | 0.04 |
| ε 3 ε 3 (Reference) | 1.00 139/1322 | 1.00 175/2786 | 1.00 314/4108 | NA |
| ε 3 ε 4 | 1.13 (0.85-1.51)  68/548 | 0.86 (0.63-1.16)  59/1083 | 0.99 (0.81-1.23)  127/1631 | 0.96 |
| ε 4 ε 4 | 0.73 (0.30-1.80)  5/49 | 1.16 (0.53-2.54)  7/97 | 0.95 (0.53-1.71)  12/146 | 0.86 |

|  | **NPHS II** | **ELSA** | **Combined studies** | **P value** |
| --- | --- | --- | --- | --- |
| *APOE* Haplotype | Unadjusted HR (95% CI) | Unadjusted OR (95% CI) | Unadjusted HR (95% CI) |  |
| ε3 ε 3 | 1.00 | 1.00 | 1.00 |  |
| ε 2+ | 0.66 (0.43-1.00) | 0.93 (0.64-1.34) | 0.80 (0.61-1.06) | P=0.23 |
| ε 4+ | 1.09 (0.82-1.45) | 0.88 (0.66-1.18) | 0.98 (0.80-1.20) | P=0.86 |
