# Supplementary material for: Circulating Apolipoprotein E Concentration and Cardiovascular Disease Risk: Meta-analysis of Results from Three Studies
Source: PLoS Med. 2016 Oct 18;13(10):e1002146. doi: 10.1371/journal.pmed.1002146 (PMC5068709; doi:10.1371/journal.pmed.1002146)
Supplement: S1 Text — (DOC) [file pmed.1002146.s007.doc]

STROBE Statement—checklist of items that should be included in reports of observational studies

|  | Item No | Recommendation |
| --- | --- | --- |
| **Title and abstract** | 1 | (*a*) Indicate the study’s design with a commonly used term in the title or the abstract  “**Large scale analysis of circulating apolipoprotein E concentration and cardiovascular disease risk, measures and meta-analysis of 3 new studies** |
|  |  | (*b*) Provide in the abstract an informative and balanced summary of what was done and what was found.  This is included, a summary of the types of study, the analyses carried out and a summary of the main findings are included |
| Introduction | | |
| Background/rationale | 2 | Explain the scientific background and rationale for the investigation being reported  This has been explained, and the rationale of this study has been placed in the context of what is known before. “To date, few studies have reported the association of circulating ApoE concentration with cardiovascular disease (CVD) events (i.e. stroke and CHD). …... We therefore aimed to examine the association of ApoE concentration with CVD events in three new studies of around 10,000 middle-aged individuals in total, with a wider range of measures of cardiovascular risk factors and 1192 CVD events.” |
| Objectives | 3 | State specific objectives, including any prespecified hypotheses  Our objectives are clearly stated in the introduction and include investigation of the association of ApoE concentrations with CVD outcomes and also describe the relationship of circulating ApoE with other measured markers present in the studies used. This has also been included in an analysis plan that was drawn up prior to analyses and is included with the submission |
| Methods | | |
| Study design | 4 | Present key elements of study design early in the paper  Methods are summarised in the abstract including the design of the studies from which new measures were made and existing data drawn “we measured circulating ApoE concentration in 9587 individuals (with 1413 CVD events) from 3 studies with incident CVD events: two population based studies, the English Longitudinal Study of Aging (ELSA) and Northwick Park Heart Study II (NPHS II), and a nested sub-study of the Anglo Scandinavian Cardiac Outcomes Trial (ASCOT). We examined the associations of circulating ApoE with cardiovascular risk factors in the two population-based studies, (ELSA and NPHS II) and the relationship between ApoE concentration and coronary heart disease and stroke in all three studies. Analyses were carried out within-study and where appropriate, pooled effect estimates were derived using meta-analysis”. These methods are expanded upon in detail in the methods section. |
| Setting | 5 | Describe the setting, locations, and relevant dates, including periods of recruitment, exposure, follow-up, and data collection  These details are all included in the description of the studies that are included- Methods paragraph 1; with references included back to the original descriptions of these studies. |
| Participants | 6 | (*a*) *Cohort study*—Give the eligibility criteria, and the sources and methods of selection of participants. Describe methods of follow-up  *Case-control study*—Give the eligibility criteria, and the sources and methods of case ascertainment and control selection. Give the rationale for the choice of cases and controls  *Cross-sectional study*—Give the eligibility criteria, and the sources and methods of selection of participants  These descriptions are included in the Methods section, paragraph 1 |
|  |  | (*b*) *Cohort study*—For matched studies, give matching criteria and number of exposed and unexposed  *Case-control study*—For matched studies, give matching criteria and the number of controls per case  ASCOT analyses was a nested case control study matching details are given “individuals matched on age, sex and country of origin was used for this study”, methods paragraph 1. |
| Variables | 7 | Clearly define all outcomes, exposures, predictors, potential confounders, and effect modifiers. Give diagnostic criteria, if applicable  Outcomes and diagnostic criteria are included again the description of the studies in summary with more detailed references to original study papers being provided. Harmonisation across study populations is highlighted too. Exposures and potential confounders are all highlighted in the baseline characteristics tables, Methods section paragraph 1. |
| Data sources/ measurement | 8* | For each variable of interest, give sources of data and details of methods of assessment (measurement). Describe comparability of assessment methods if there is more than one group.  ApoE was the main measure that was being made, and methods of measurement and the instrument the measurement was made on are included in the Methods section, paragraph 2, under the heading “*ApoE Measurement”* with the following text “Circulating ApoE was measured using a nephelometric method on a BN II Nephelopmeter (Siemens, UK), using a non-isoform specific polyclonal antibody. ApoE measures from NPHSII were made in citrated plasma taken at the fourth annual visit and were taken after a light breakfast. ELSA and ASCOT measures were made in serum. Samples used in ELSA were taken at wave 2. Most participants were fasted, those over age of 70 (35%) and diabetics (7.2%) were not asked to fast (total 35.8% of ELSA participants), although most were seen prior to eating breakfast. In ASCOT samples were taken at randomisation in the fasted state. Samples used for all measures were taken at a time prior to any CVD events. Differences in concentration dependent on whether plasma or serum was used for ApoE measurement was overcome at the analysis stage by standardising measures of ApoE”. |
| Bias | 9 | Describe any efforts to address potential sources of bias  Bias and confounding are dealt with at the analyses stage by adjustments, and because the manuscript addresses the clinical utility of a measurement, adjustments are made for the Framingham variables which are those that are in use clinically. This is described in the Methods, in the subsection entitled “Association of circulating ApoE and cardiovascular events”. |
| Study size | 10 | Explain how the study size was arrived at  Study size is given, and the numbers in of samples that were available to make the ApoE measures was maximised, and all are detailed in the methods section, throughout the relevant sections. |
| Quantitative variables | 11 | Explain how quantitative variables were handled in the analyses. If applicable, describe which groupings were chosen and why  All data handling descriptions, including log transformations and why this was carried out are included in the statistical analysis section of the manuscript. |
| Statistical methods | 12 | (*a*) Describe all statistical methods, including those used to control for confounding |
|  |  | (*b*) Describe any methods used to examine subgroups and interactions |
|  |  | (*c*) Explain how missing data were addressed |
|  |  | (*d*) *Cohort study*—If applicable, explain how loss to follow-up was addressed  *Case-control study*—If applicable, explain how matching of cases and controls was addressed  *Cross-sectional study*—If applicable, describe analytical methods taking account of sampling strategy |
|  |  | (*e*) Describe any sensitivity analyses  All methods are described in detail in the statistical analysis section, without reproducing the entire section here, adjustments for potential confounders pertaining to clinical utility are included, correlations are described both numerically and graphically, follow up was addressed using Cox proportional methods and logistic regression for case control subsets. Matching for the ASCOT study is already described. |

| Results | | |
| --- | --- | --- |
| Participants | 13* | (a) Report numbers of individuals at each stage of study—eg numbers potentially eligible, examined for eligibility, confirmed eligible, included in the study, completing follow-up, and analysed  These are detailed in results tables and the text throughout the results and supporting information. Proportions of missing data and how these were handled are also included. |
|  |  | (b) Give reasons for non-participation at each stage NA |
|  |  | (c) Consider use of a flow diagram These are described in detail in the text |
| Descriptive data | 14* | (a) Give characteristics of study participants (eg demographic, clinical, social) and information on exposures and potential confounders  Detailed in the baseline Table 1 |
|  |  | (b) Indicate number of participants with missing data for each variable of interest  Proportions of missing data and how these were handled are included in the methods |
|  |  | (c) *Cohort study*—Summarise follow-up time (eg, average and total amount) |
| Outcome data | 15* | *Cohort study*—Report numbers of outcome events or summary measures over time  Included in Tables in the main and supporting information |
|  |  | *Case-control study—*Report numbers in each exposure category, or summary measures of exposure  Included in Tables in the main and supporting information |
|  |  | *Cross-sectional study—*Report numbers of outcome events or summary measures NA |
| Main results | 16 | (*a*) Give unadjusted estimates and, if applicable, confounder-adjusted estimates and their precision (eg, 95% confidence interval). Make clear which confounders were adjusted for and why they were included  These are all detailed both in methods, that is which confounders would be adjusted for and are detailed in the main text of the results section as well as graphically outlined in the Figures. |
|  |  | (*b*) Report category boundaries when continuous variables were categorized  Tertiles of ApoE are given and these are detailed in the Tables of results. No other categorisations were included |
|  |  | (*c*) If relevant, consider translating estimates of relative risk into absolute risk for a meaningful time period NA |
| Other analyses | 17 | Report other analyses done—eg analyses of subgroups and interactions, and sensitivity analyses NA |
| Discussion | | |
| Key results | 18 | Summarise key results with reference to study objectives This is included in the first paragraph of the discussion |
| Limitations | 19 | Discuss limitations of the study, taking into account sources of potential bias or imprecision. Discuss both direction and magnitude of any potential bias  There is a section of limitations in the discussion that details this |
| Interpretation | 20 | Give a cautious overall interpretation of results considering objectives, limitations, multiplicity of analyses, results from similar studies, and other relevant evidence  As above, the findings are placed in the context of what is known and interpretations also take into account limitations |
| Generalisability | 21 | Discuss the generalisability (external validity) of the study results  Discussed in detail towards the end of the discussion, and the need for additional work and what kind of work this might be |
| Other information | | |
| Funding | 22 | Give the source of funding and the role of the funders for the present study and, if applicable, for the original study on which the present article is based  Funders for all co-authors is detailed |

*Give information separately for cases and controls in case-control studies and, if applicable, for exposed and unexposed groups in cohort and cross-sectional studies.

**Note:** An Explanation and Elaboration article discusses each checklist item and gives methodological background and published examples of transparent reporting. The STROBE checklist is best used in conjunction with this article (freely available on the Web sites of PLoS Medicine at http://www.plosmedicine.org/, Annals of Internal Medicine at http://www.annals.org/, and Epidemiology at http://www.epidem.com/). Information on the STROBE Initiative is available at www.strobe-statement.org.
