# Supplementary material for: Circulating Apolipoprotein E Concentration and Cardiovascular Disease Risk: Meta-analysis of Results from Three Studies
Source: PLoS Med. 2016 Oct 18;13(10):e1002146. doi: 10.1371/journal.pmed.1002146 (PMC5068709; doi:10.1371/journal.pmed.1002146)
Supplement: S2 Text — (DOC) [file pmed.1002146.s008.doc]

**ApoE genotype and levels and risk of coronary heart disease: Statistical analysis plan January 2015**

Analysis:

1. Baseline characteristics: Mean and standard deviations should be presented for normally distributed continuous variables and percentages for binary variables. Non-normally distributed continuous variables (apoE, CRP, TRG for example) should be log transformed using natural logs prior to any analyses and geometric means with approximate SD’s calculated. Tables for all studies to be completed and can be reported in supplementary section
2. Univariate associations of circulating ApoE with measured markers in each study, assessed using correlation coefficient, both age adjusted and unadjusted.
3. Association of biomarkers and traits with ApoE levels as a categorical variable divided into tertiles in order to describe more clearly the relationship of ApoE with other markers. Report in tables with p trend. Visualise these associations using graphical means, dividing markers into deciles and plotting against ApoE so shape of distribution can be determined.
4. Association of ApoE levels with CVD using the following categories where these are available in this way across all studies.

- CHD-ALL (fatal and non fatal), CHD-FATAL, CHD-NON FATAL
- STROKE-ALL (fatal and non fatal), STROKE-FATAL, STROKE-NON FATAL (check if sub-categories of stroke are available and if analyses are possible in this way)
- CVD-FATAL (CHD AND STROKE FATAL), CVD-NON FATAL (CHD AND STROKE NON FATAL)

Pool outcomes across all studies using meta-analysis, using a random effects model, weighting effect size by the inverse of the variance and assessing heterogeneity using Der Simonian and Laird Q test and quantified using an I^2^ statistic.

1. For prospective studies, Cox proportional hazards models should be used. All co-variates should be standardised, data set as survival data, and then models constructed. A priori hypothesis that we are trying to test is if ApoE concentration is associated with CVD and could it have a clinical use in prediction. This would be most usefully analysed using Framingham model and adding ApoE as an additional variable in this multivariate model. However given genetic data have indicated that the effect of APOE genotype may be mediated through LDL-C, consider this in the model. Therefore 4 models defined a priori, crude, adjusted by age and sex, ApoE +Framingham and ApoE+Framingham + LDL-C. Primary model should be based on CVD risk per SD increase in log ApoE although secondary analyses to seek the risk of CVD per tertile explored with p value for trend across tertiles.

There is an argument to use all variables that are available and comparable across studies, given the potential confounders, and could be informed from cross sectional analyses. However how this could then be of clinical utility over and above Framingham is uncertain.

1. For case-control studies odds ratios will be calculated using conditional logistic regression models. As above models with no adjustments, adjustments for Framingham covariates and adjustments for Framingham in addition to other significant covariates will be calculated.
2. Time to event analyses: for prospective studies (ELSA and NPHS II) add Kaplan Meier plots, stratifying by concentration of ApoE, that is using ε3 ε3 as the baseline group and comparing other groups to this.

**Additional analyses carried out and added to final manuscript through review process and editorial comments**

1) Analysis of ApoE association with CVD by quintiles and additional quadratic model to demonstrate the absence of a U shaped effect as an explanation of a lack of association between ApoE and CVD.

2) Removal of Model 4, Framingham and LDL-C adjusted because of co-linearity

3) Removal of ELSA from survival analyses because of period of time rather than exact time of event being available.

2) Use of CRP regression dilution ratios as a sensitivity analysis to estimate regression dilution bias that may cause the attenuation in the size of the observed effect between ApoE and CVD.

3) Power calculation of the genetic effect of *APOE* on CVD in ELSA and NPHSII compared to large scale existing studies
